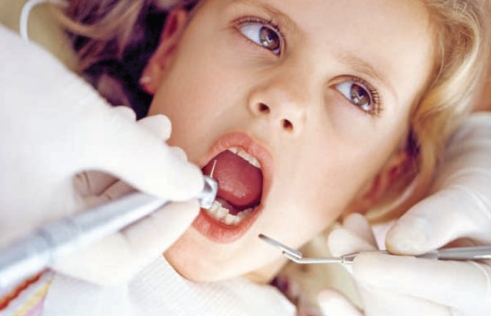# Taking a Bite Out of Amalgam Concerns?: Study Shows No Renal Effects in Children

**DOI:** 10.1289/ehp.116-a129a

**Published:** 2008-03

**Authors:** Victoria McGovern

Dental amalgam is a major source of human exposure to inorganic mercury, which is thought to occur primarily when elemental mercury from the amalgam surface evaporates and is inhaled. Prior studies provide strong evidence that the central nervous system and the kidney are the primary targets of inorganic mercury. Data from the New England Children’s Amalgam Trial (NECAT), a clinical trial designed to study the possible health effects in children of mercury-containing dental amalgam, now indicate that amalgam fillings’ effects on renal function may be quite small **[*EHP* 116:394–399; Barregard et al.]**.

The study, launched in 1996 in Maine and Massachusetts, is one of two parallel randomized trials funded by the National Institute of Dental and Craniofacial Research. These trials provide the first rigorously designed clinical data on the effects of children’s dental exposure to mercury, a known neuro- and nephrotoxicant.

A group of 537 children aged 6–10 years at the start of the trial were followed for five years. Children began the trial with no pre-existing fillings and at least one cavity in a back tooth. The children were randomly assigned to two groups, one receiving only amalgam fillings in cavities in the back teeth and the other receiving only composite fillings in the back teeth. Both groups received the more aesthetically pleasing composite fillings in the front teeth, conforming to current standard dental practice.

The children showed no statistically significant differences in several markers of toxicity to renal tubules studied—including *N*-acetyl-β-D-glucosaminidase and alpha-1-microglobulin, both early indicators of toxic exposure to mercury vapor, or in γ-glutamyl transpeptidase, which has been shown to be affected by toxic heavy metal exposure—regardless of whether their cavities were filled with amalgam or with composite.

Microalbuminuria, the occurrence of small quantities of albumin in the urine, increased in 10 children treated with amalgam and in 2 treated with composite fillings, but it did not correlate with the number of amalgam fillings or with increasing concentrations of mercury excreted in urine.

Because this difference is marginally significant, the authors note it may be due to chance. Moreover, microalbuminuria can occur transiently as a consequence of recent vigorous play. The results therefore appear to indicate that the use of amalgam fillings does not cause any clear, consistent damage to kidney function in young, developing children.

## Figures and Tables

**Figure:**